# Correction: Stability of spontaneous, correlated activity in mouse auditory cortex

**DOI:** 10.1371/journal.pcbi.1010232

**Published:** 2022-06-06

**Authors:** Richard F. Betzel, Katherine C. Wood, Christopher Angeloni, Maria Neimark Geffen, Danielle S. Bassett

S1 and S2 Figs are corrupted on the published article. The authors have provided corrected versions here.

## Supporting information

S1 Fig Module similarity at different hierarchical levels. (TIF)

S2 Fig Examples of cell tracking over days. (TIF)
