# Supplementary figures and images for: Correction: Stability of spontaneous, correlated activity in mouse auditory cortex
Source: PLoS Comput Biol. 2022 Jun 6;18(6):e1010232. doi: 10.1371/journal.pcbi.1010232 (PMC9170108; doi:10.1371/journal.pcbi.1010232)

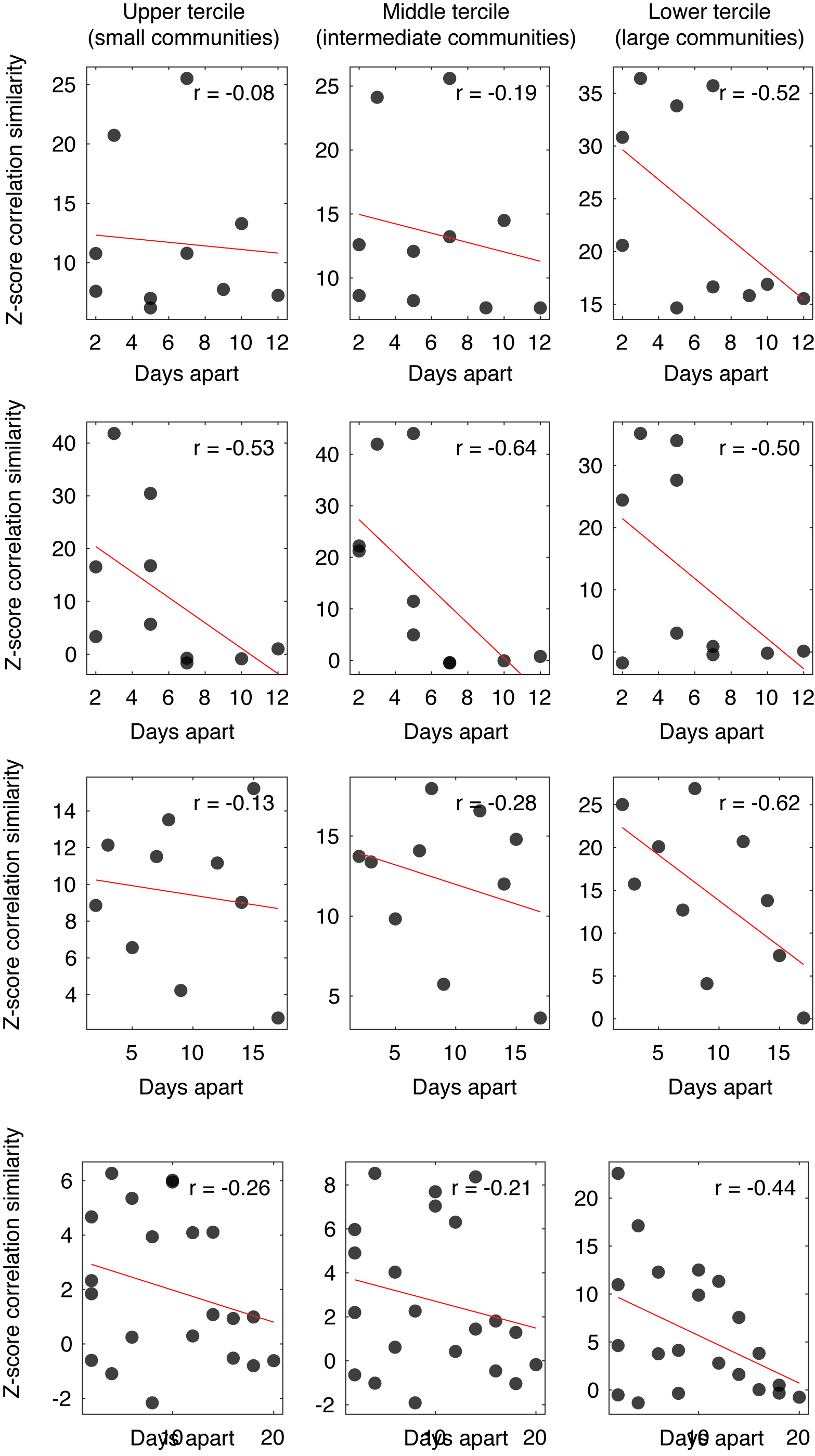

Supplement: S1 Fig — (TIF) [file pcbi.1010232.s001.tif]

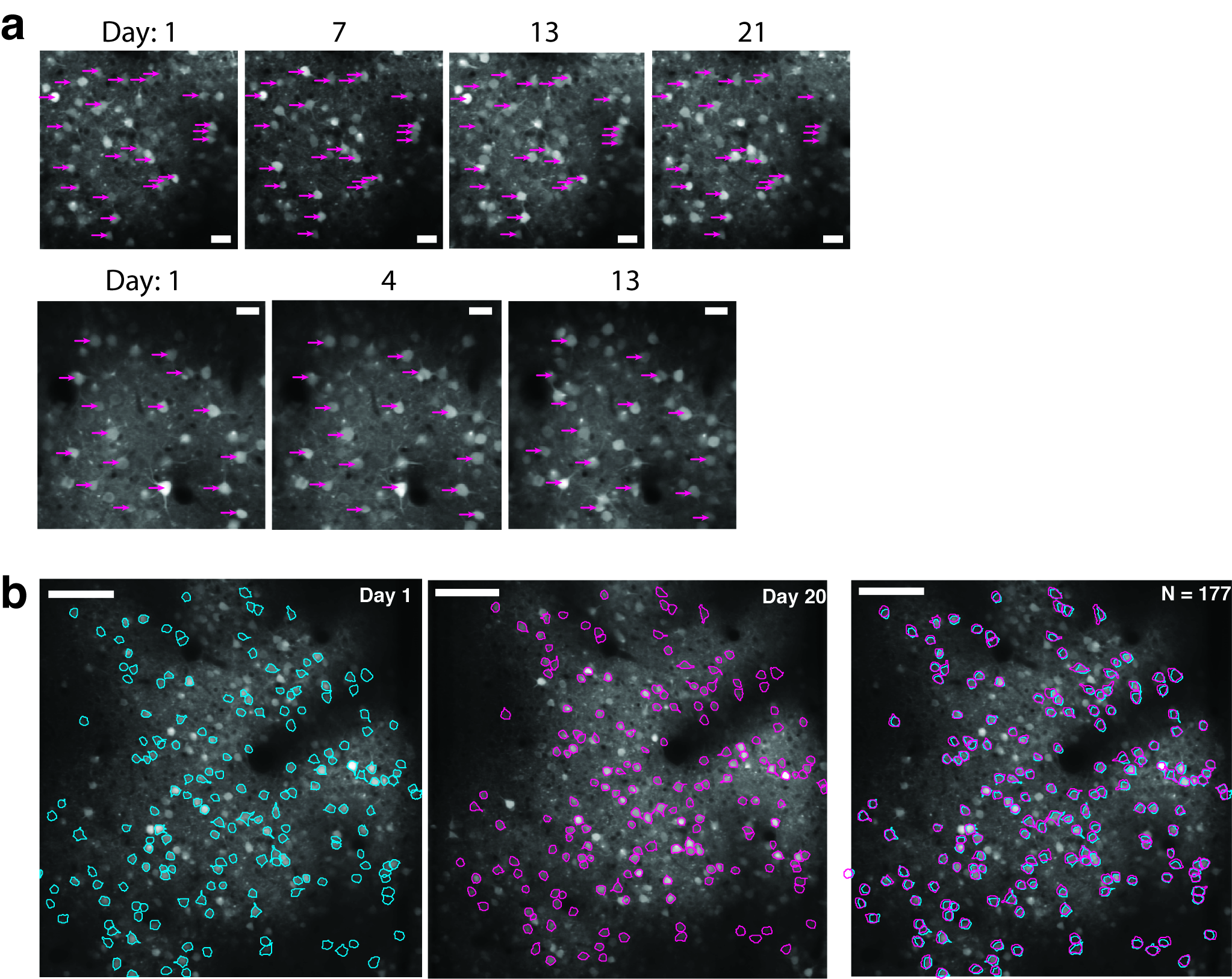

Supplement: S2 Fig — (TIF) [file pcbi.1010232.s002.tif]
